# A Rare Case of Acute Liver Failure Secondary to Miliary Liver Metastasis

**DOI:** 10.7759/cureus.32282

**Published:** 2022-12-07

**Authors:** Prabasha Weeraddana, Teena Thomas, Niwanthi Weerasooriya, Mohamed Zakee Mohamed Jiffry, Dmitriy Golovyan, Shubhneet Bal

**Affiliations:** 1 Internal Medicine, Danbury Hospital, Danbury, USA; 2 Pulmonary and Critical Care, Danbury Hospital, Danbury, USA; 3 Pathology, Danbury Hospital, Danbury, USA

**Keywords:** ct abdomen, alt (alanine aminotransferase), ast (aspartate aminotransferase), non- small cell carcinoma, liver metastasis, acute liver failure (alf), miliary metastasis

## Abstract

Acute liver failure (ALF) is a potentially fatal condition that adversely affects multiple organs and has a high mortality rate. ALF due to hepatic infiltration is rare even though it is a common place for cancer to metastasize. Patients with ALF usually present with abdominal pain and elevated liver function tests. We report a case of a 65-year-old male that developed ALF due to miliary liver metastasis of non-small cell carcinoma from an unknown primary origin. The patient initially presented with a couple of episodes of coffee-ground emesis and epigastric pain. Upon further evaluation, along with computed tomography (CT) scans and liver biopsy, the diagnosis was established. The patient passed away on the 12^th^ day of hospitalization.

## Introduction

Acute liver failure (ALF), also known as fulminant hepatic failure, is a clinical phenomenon characterized by severe acute liver injury with encephalopathy and impaired synthetic function (international normalized ratio (INR) >1.5) in a patient without the pre-existing liver disease [1]. ALF has a variety of causes that include both primary causes as well as secondary ones. The primary causes include viruses and intrinsic liver disease. However, ALF can be secondary to drugs and cancer metastasis [2]. Encephalopathy, cerebral edema, sepsis, renal failure, and respiratory failure are some possible complications of liver failure [3]. There were some instances that liver imaging studies that were nondiagnostic and the malignant liver infiltration was confirmed at postmortem [1]. A proper diagnosis by liver biopsy is essential but may fail to recognize the primary site of origin as in our case. Effective chemotherapy has improved the survival of patients with metastatic liver disease. But it’s challenging to initiate such treatment on these patients as most of the patients are unstable and severely ill with multi-organ failure at the time of final diagnosis.

## Case presentation

A 65-year-old male presented to the emergency department (ED) with two episodes of coffee-ground emesis, epigastric pain, generalized weakness, and poor oral intake. He also had an episode of fever a week ago associated with chills and rigors that lasted for five to six days. The fever resolved spontaneously with Tylenol intake. A week prior, he started experiencing epigastric abdominal pain, which was dull and non-radiating. The pain was five out of ten in severity, which increased on the day of admission, after the episode of coffee-ground emesis. The patient denied any upper respiratory symptoms or urinary symptoms. He has had a weight loss of about 10 lbs over the past 10 days which he thought was due to poor oral intake. He also noted that his urine color is very dark. He is unable to keep liquids down due to nausea. On the day of admission, in the morning, he vomited dark brown vomitus, which prompted him to present to the ED.

The patient is a current smoker of 60 packs a year and had a medical history of anxiety, gastroesophageal reflux disease (GERD), irritable bowel syndrome, and chronic constipation. He does have a family history of colon cancer. The patient had several unsuccessful colonoscopies due to poor preparation. There was no history of hepatotoxic drugs and natural or herbal products consumption. He drinks alcohol occasionally. On admission he was afebrile, tachycardic (102-110 per minute), and hypotensive at 79/57 mmHg.The patient appears cachectic, dehydrated, and with an icteric sclera. Air entry was present bilaterally without rhonchi and bilateral lower extremity edema was noted. Epigastric tenderness was present and bowel sounds were quite audible but there were no other features of chronic liver disease or hepatomegaly. There were no palpable lymph nodes in the cervical/supraclavicular/inguinal/axillary region. Blood workup shows total bilirubin 6.6 mg/dL, direct bilirubin 5 mg/dL, gamma-glutamyl transferase (GGT) 415 U/L, alanine aminotransferase (ALT) 220 U/L, aspartate aminotransferase (AST) 429 U/L, lipase 15 U/L. The complete blood count revealed leukocytosis with a white blood cell count of 33.4 × 109/L, predominantly neutrophilic. His hemoglobin was 11.3 g/dL and his platelet count was 605 × 109/L. Procalcitonin was 5.5 ng/mL (elevated), INR 1.23, PT 15.6 seconds, and activated partial thromboplastin time (APTT) 33.5 seconds while acetaminophen level in the blood was < 5 mcg/mL. The patient was tested non-reactive for Hepatitis A, B, and C. The tick panel was negative. 

**Figure 1:**
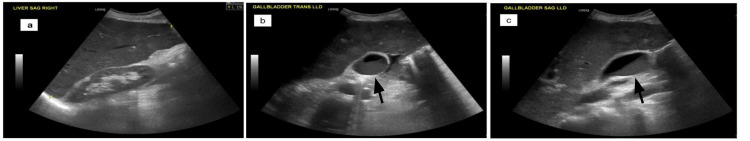
Ultrasound scan of the right upper quadrant of the abdomen ( a- liver in the sagittal plane, b- gallbladder in the transverse plane, c- liver and gallbladder in the sagittal plane,) showing layering sludge within an otherwise normal-appearing gallbladder (black arrow) and the liver demonstrates the heterogeneity of its parenchyma and nodularity of its contour.

**Figure 2:**
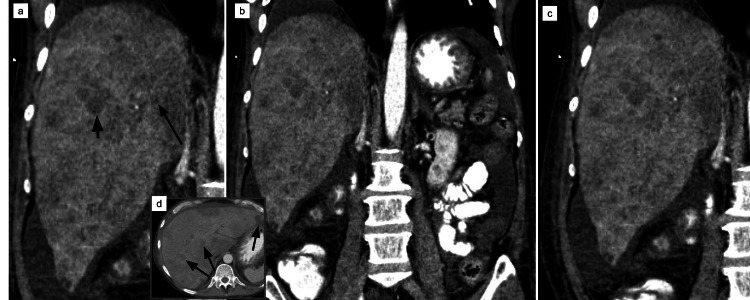
computed tomography (CT ) abdomen and pelvis with contrast (a,b,c - coronal view, d- axial view ) showing diffused innumerable hypodense nodules involving both lobes of the liver in various plains. The thick arrow denotes the largest and the thin arrows smaller nodules.

**Figure 3:**
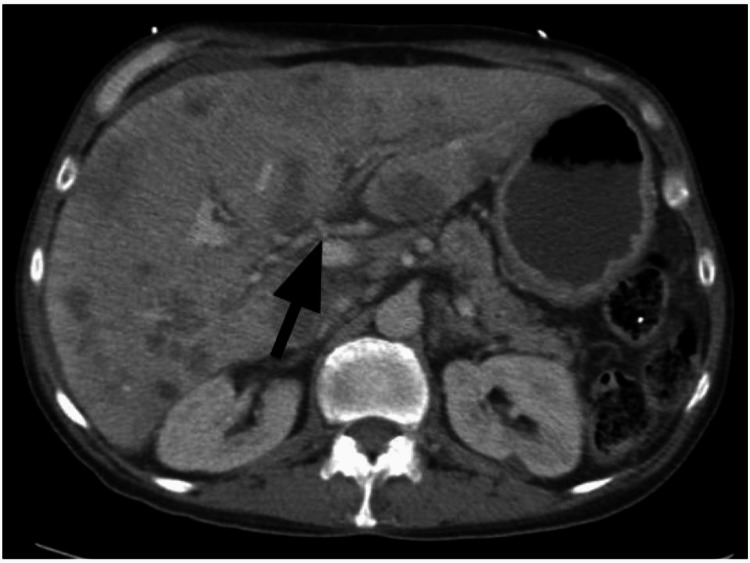
The computed tomography (CT) abdomen and pelvis with contrast revealed a partial left portal vein filling defect suggestive of thrombosis(black arrow).

**Figure 4:**
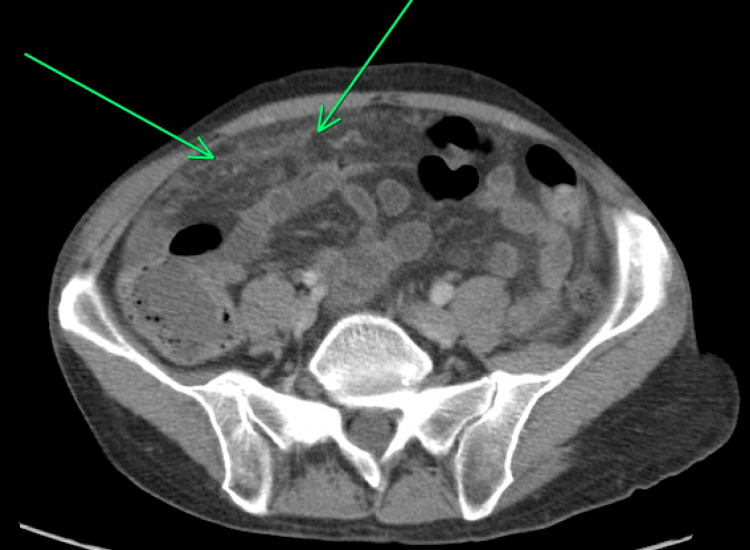
computed tomography (CT) abdomen and pelvis with contrast prominent nodularity of the mesentery (green arrows) which may represent venous congestion from portal hypertension versus peritoneal carcinomatosis.

An ultrasound scan of the right upper quadrant of the abdomen revealed biliary sludge. It further demonstrated heterogeneity of the liver parenchyma and nodularity of its contour, which was suggestive of cirrhosis (Figure 1). The computed tomography (CT) abdomen and pelvis with contrast revealed the extent of miliary liver metastases (Figure 2), with a partial left portal vein filling defect suggestive of thrombosis (Figure 3). There is prominent nodularity of the mesentery which may represent venous congestion from portal hypertension versus peritoneal carcinomatosis (Figure 4).

In the ED, the patient received normal saline boluses, intravenous (IV) empiric cefepime and metronidazole, and Zofran. The patient was initially admitted to the progressive care unit (PCU) with a working diagnosis of direct hyperbilirubinemia in the setting of metastatic disease from an unknown primary versus decompensated cirrhosis with portal vein thrombosis complicated by septic shock.

The etiology of septic shock could be explained in the setting of cholangitis. However, other possibilities like pneumonia cannot be ruled out given elevated procalcitonin (5.45 ng/mL). The patient was continued on broad-spectrum antibiotics, including cefepime and metronidazole. Phenylephrine was started to maintain blood pressure. The next day, the patient had another episode of coffee-ground emesis, and his hemoglobin dropped to 8.1 g/dL. Therefore, he received two units of packed red blood cells and was started on intravenous pantoprazole 40 mg twice daily. His blood culture revealed coagulase-negative Staphylococcus in two out of four bottles. The patient underwent a mesenteric vein duplex scan and portal vein thrombosis was excluded. 

**Figure 5:**
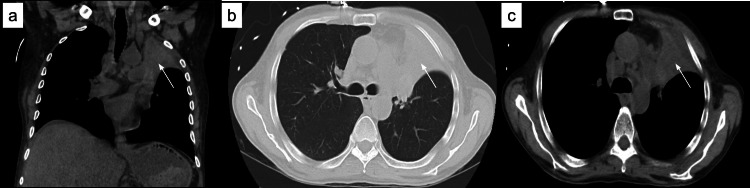
computed tomography (CT) chest without contrast revealed obstruction of the left upper lobe bronchus with the complete collapse of the left upper lobe (white arrow). (a-coronal view, b and c - axial view).

**Figure 6:**
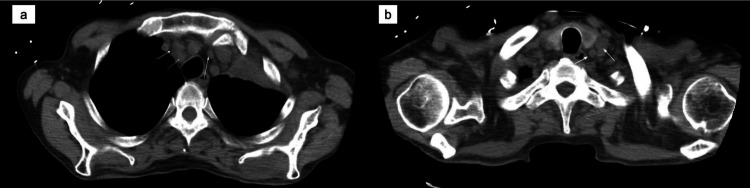
computed tomography CT chest showing mediastinal (a) and left supraclavicular lymphadenopathy(b) which is likely metastatic.

Meanwhile, the patient also underwent a chest CT without contrast as he developed shortness of breath. The CT chest showed obstruction of the left upper lobe bronchus with the complete collapse of the left upper lobe (Figure 5). The cause of the obstruction was suspected to be either left hilar lymphadenopathy or an endobronchial mass. Mediastinal and left supraclavicular lymphadenopathy were suspected to be metastatic (Figure 6). 

His CEA was elevated to 1289 ng/mL while his AFP was 5.9 ng/mL. He received another two units of packed red blood cells (PRBCs) as his Hb level further dropped to 7.5g/dL.

On post-hospitalization day four, CT of the chest and abdomen was repeated to look for the source of sepsis from an intra-abdominal occult abscess. It showed stable upper lobe collapse of the left lung but no evidence of an abscess of the abdomen or pelvis. He was able to wean off from pressure support and stabilize hemoglobin levels to around 10 g/dL. On day seven, the patient was transferred to the general medical floor. The next day, the patient underwent an ultrasound-guided needle biopsy of the liver. The plan was to pursue further work-up with bronchoscopy if the gastrointestinal workup was negative.

**Figure 7:**
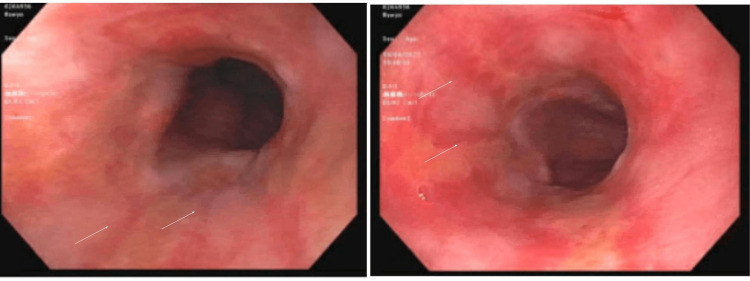
Esophagogastroduodenoscopy (EGD) shows a grade B esophagitis (indicated by white arrows) with no bleeding ulcers was found in the distal esophagus.

**Figure 8:**
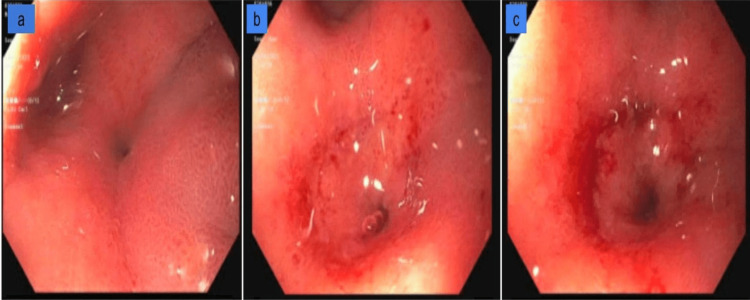
esophagogastroduodenoscopy (EGD) shows benign-appearing, intrinsic severe stenosis at the pylorus (a-pre-pyloric stenosis, b and c - pyloric stenosis).

The next day, the patient underwent esophagogastroduodenoscopy (EGD), which showed grade B esophagitis (one or more mucosal breaks greater than 5 mm, not extending between the tops of two mucosal folds) with no bleeding ulcers (Figure 7). A benign-appearing, intrinsic severe stenosis was found at the pylorus which was dilated using a balloon dilator (Figure 8). The gastric body, antrum, and duodenum appear normal. Biopsies were taken with cold forceps which were negative for malignancy and H. pylori. Also, there was no sign of metaplasia (Barrett’s esophagus).

**Figure 9:**
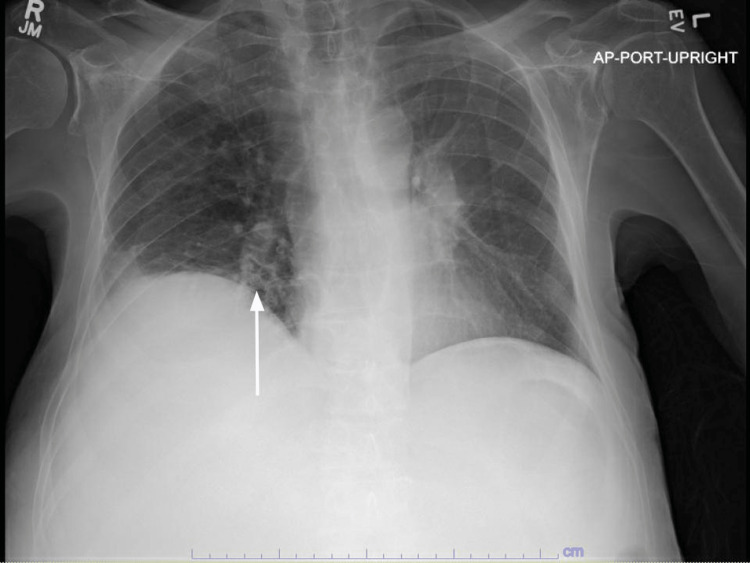
Chest XR portable showed right lung base opacities, which was concerning for atelectasis and pneumonia (white arrow).

On post-admission day 11, the patient's clinical status began to worsen. He developed labored breathing using accessory muscles and he became confused. Examination showed rhonchi throughout and crackles with decreased breath sounds at the right base. The patient was disoriented in time and space and was talking about irrelevant things. The venous blood gas (VBG) revealed a pH of 7.11. The chest X-ray showed right lung base opacities, which was concerning for atelectasis and pneumonia (Figure 9). His blood potassium level was elevated to 7.1 mmol/L, bicarbonate levels were 6 mEq/L (significantly low), and the anion gap was 22 mEq/L (elevated). 

His creatinine levels were also elevated to 1.66 mg/dL. Moreover, his leukocytes were 32.6 0 × 109/L (leukocytosis), lactic acid 10.4 mg/dL, INR 4.16, APTT 62.3 seconds, and PT 39.5 seconds. His liver function test (LFT) showed total bilirubin 11.9 mg/dL, direct bilirubin 9.9 mg/dL, alkaline phosphatase (ALP) 524 U/L, ALT 251 U/L, AST 957 U/L. He was then started on IV calcium gluconate, insulin, and dextrose for hyperkalemia. The day-to-day blood workup is shown in Table 1. 

**Table 1:** DAY-TO-DAY BLOOD WORKUP (DAY 0 BEING THE DAY OF ADMISSION) ALP alkaline phosphatase, GGT gamma-glutamyl transferase, ALT: alanine aminotransferase, AST: aspartate aminotransferase, LDH- lactate dehydrogenase

TEST NAME	DAY 0	DAY 1	DAY 2	DAY 5	DAY 6	DAY 7	DAY 8	DAY 9	DAY 10	DAY 11	DAY 12
PHOSPHORUS (mg/dL)	-	2.3	-	-	2.1	2	3.2	-	-	-	-
MAGNESIUM (mg/dL)	2.3	2.1	-	-	2.1	2	-	-	-	-	-
ALBUMIN (g/dL)	2.6	2	-	-	-	-	-	-	-	-	-
BILIRUBIN TOTAL (mg/dL)	6.6	4.8	4.8	5.6	7	9.8	10.3	10.7	11.9	9.9	15.7
BILIRUBIN DIRECT (mg/dL)	5	4.1	4.2	4.7	5.7	7	8.1	9.2	9.9	8	-
ALP (IU/L)	737	590	492	461	430	464	418	545	524	458	-
GGT (U/L)	415	-	-	-	-	-	-	-	-	-	-
ALT (U/L)	220	135	120	115	141	169	159	252	251	317	-
AST (U/L)	429	253	217	244	501	-	444	1091	957	1918	-
LDH (IU/L)	946	-	-	-	-	-	-	-	-	-	-
LIPASE (U/L)	15	-	-	-	-	-	-	-	-	-	-

Subsequently, the patient's systolic blood pressure trended down to 70 mmHg. The patient was transferred to the intensive care unit (ICU) for further management. Due to increased work of breathing and shock, the patient was intubated urgently. Prior to the intubation attempt, he vomited a copious amount of coffee ground emesis. His nasogastric tube drained around one liter(L) of blood post-intubation. The patient received a total of three liters of normal saline. He was started on Levophed as he continued to be hypotensive. He also received two units of PRBC given hemodynamic instability. There was a continuous suction of coffee-ground fluid from his stomach. His acute respiratory failure was likely secondary to hospital-acquired pneumonia (HAP) versus aspiration pneumonia. The patient also had an endobronchial lesion along with obstruction in the right upper lobe bronchus with the complete collapse of the right upper lobe in the CT chest, which can be contributory. He had acute liver failure along with multiple organ system failures, including respiratory, renal, and cardiovascular systems.

**Figure 10:**
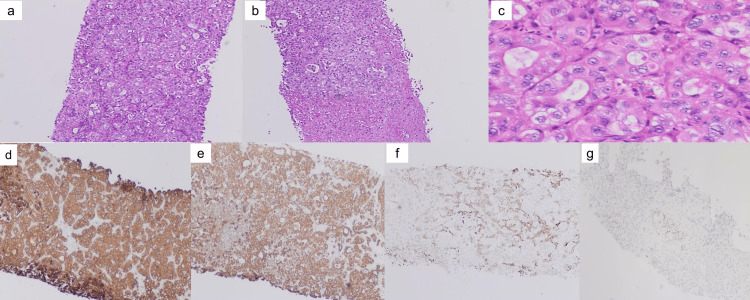
The H & E stained sections (a and C) from ultrasound-guided core needle biopsy shows liver tissue infiltrated by malignant epithelial cells that are round to polygonal with an enlarged nucleus, vesicular chromatin, and prominent nucleoli. The malignant cells make a focal attempt at gland formation with some areas of normal-appearing hepatocytes in the background. The H&E stained section(b) shows areas of necrosis. Immunostains of liver biopsy (d to e): The malignant cells are positive for cytokeratin AE 1/3 (d), cytokeratin 7(e), and weak patchy positive for cytokeratin 20 (f) and CDX2 (e), supporting the diagnosis.

On the 12th day of hospitalization, the pathology report of his liver biopsy established the diagnosis of metastatic non-small cell carcinoma favoring adenocarcinoma (Figure 10).

Immunostains of the liver biopsy were positive for Cytokeratin 7, Cytokeratin AE ⅓, weak patchy positive for Cytokeratin 20 and CDX2, negative for TTF1, Napsin A, NKX 3.1, PSA, HepPar-1 and Glypican.

The immunoprofile of the biopsy was supportive of the metastatic poorly differentiated non-small cell carcinoma. However, a definitive primary site for the carcinoma could not be ascertained. Potential primary sites included upper GI, pancreatobiliary, and lung, but other sites could not be excluded. After a discussion with the family, the decision was made to extubate the patient and switch to a comfort pathway. The patient passed away after palliative extubation on the same day.

## Discussion

Acute liver failure (ALF) is a rare but life-threatening condition that causes multiorgan effects [1]. Although the liver is the most frequent site for metastasis (36%), ALF from malignant infiltration is extremely rare [4]. Due to the rarity of such case presentations, the literature on the topic is also limited. Furthermore, the aspect of multiorgan failure in the condition makes the management of such patients challenging. It has also been found that the diagnosis of diffuse hepatic infiltration is difficult to make in imaging studies [5].

Most reported cases of ALF due to infiltration of metastatic carcinoma were identified in the post-mortem studies. Currently, a liver biopsy is the best approach to make a diagnosis. However, coagulopathy associated with ALF because of INR ≥1.5 makes the procedure quite challenging. Rich et al. report that only 27 (1.4%) of the 1910 cases of lymphoma in the US ALF study group analysis had ALF due to malignant infiltration, Rowbotham and associates analyzed 4,020 ALF cases over 18 years and identified only 18 with ALF attributable to hepatic infiltration, from that only four were reported due to infiltrative metastatic carcinoma, which shows the rarity of such case presentations [6-7].

The levels of liver enzymes also hold special importance in making a diagnosis of ALF due to hepatic infiltration of metastatic cancer from an unidentified primary origin. Rowbotham et al. conclude that serum transaminase levels (median AST 358, range 78-768 IU/L) may not be very high compared to the typical extreme elevation in non-infiltrative conditions leading to ALF [7]. 

Miliary metastases are diffuse, numerous, and tiny metastatic lesions (usually less than 1 cm) involving more than one organ lobe and bilaterally distributed [8]. Epidermal growth factor receptor mutations in non-small-cell lung carcinoma, BRCA1/2 mutation in ovarian cancer, and E-cadherin mutations in breast carcinoma are typically associated with the miliary pattern of metastasis [8-10]. One of the mechanisms proposed by Allison KH et al. for diffuse infiltration of metastasis cancers includes inactivation of the E-cadherin system which is important in cell-to-cell adherence leading to cancer cells detached from primary cancer, invading surrounding tissues, entering the circulation, spreading to the distant target organ and finally proliferate [11]. Meanwhile, CD44 is responsible for endothelium adhesion. In the absence of its expression, a carcinoma cell may not be able to invade endothelial cells to create large metastatic lesions instead it diffusely infiltrates hepatic sinusoids [11].

Our case is a 65-year-old male that presented to the ED with a complaint of epigastric pain and a couple of episodes of coffee ground emesis. The patient was a current smoker. A blood workup revealed elevated liver enzymes, i.e., ALT, AST, etc. Further study and repeated ultrasounds and CT scans of the chest and abdomen showed hilar lymphadenopathy. It was suspected to be a case of miliary metastases with the liver as a site of metastasis. Later, the patient’s liver was biopsied, which established the diagnosis of acute liver failure due to miliary metastasis of non-small cell carcinoma favoring adenocarcinoma of unknown primary origin. Such a case of ALF secondary to metastatic cancer is rare. The management of such a case is quite challenging due to organ dysfunction or poor performance status.

ALF due to liver infiltration of active cancer is a contraindication for liver transplantation. Interestingly there were few cases reported who underwent liver transplants with metastatic liver disease (unrecognized before the transplant ) and subsequent chemotherapy, which did indeed do well in terms of prolonged survival [6]. Effective chemotherapy has improved the survival of patients with metastatic liver disease. But it’s challenging to initiate such treatment on these patients as most of the patients are critically ill with multi-organ failure at the time of final diagnosis. 

## Conclusions

To conclude, the case presented represents a rare case of ALF due to miliary metastasis of non-small cell carcinoma of unknown primary origin. To the best of our knowledge, this is the second reported case of such a presentation. A proper diagnosis by liver biopsy is very important but may fail to recognize the primary site of origin as in our case. Even though in our case we recognized the liver metastasis by imaging, the patient's condition deteriorated very rapidly even before starting treatment, emphasizing the need for further research and studies on improving mortality and survival of such cases.
